# Conserved phylogenetic distribution and limited antibiotic resistance of class 1 integrons revealed by assessing the bacterial genome and plasmid collection

**DOI:** 10.1186/s40168-018-0516-2

**Published:** 2018-07-21

**Authors:** An Ni Zhang, Li-Guan Li, Liping Ma, Michael R. Gillings, James M. Tiedje, Tong Zhang

**Affiliations:** 1 Environmental Biotechnology Laboratory, The University of Hong Kong, Hong Kong, China; 2 Department of Biological Sciences, Species Spectrum Research Centre, Macquarie University, Sydney, New South Wales Australia; 3 Center for Microbial Ecology, Michigan State University, East Lansing, MI USA; 4 International Center for Antibiotics and Resistance in Environments, Southern University of Science and Technology, Shenzhen, China

**Keywords:** Class 1 integrons, Antibiotic resistance, Whole genome analysis, Bioinformatics, Database construction

## Abstract

**Background:**

Integrons, especially the class 1 integrons, are major contributors to the acquisition and dissemination of antibiotic resistance genes (ARGs). However, comprehensive knowledge of the types, content, and distribution of integrons in bacterial taxa is lacking to evaluate their contribution.

**Results:**

We have constructed a new integrase database and developed a pipeline that provides comprehensive recovery of class 1 integrons. Previous PCR-based techniques might only detect one fourth of the integron-integrases and integrons recovered in this study. By exploring the class 1 integrons in over 73,000 currently available complete and draft bacterial genomes, the contribution of class 1 integrons in spreading and acquiring ARGs was evaluated. Firstly, the host species of class 1 integrons are highly conserved within (96%) in class *Gammaproteobacteria*, dominated by four pathogenic species of “ESKAPE.” Secondly, more than half of class 1 integrons are embedded in chromosomes with less potential for horizontal gene transfer. Finally, ARGs that have been acquired by these integrons only cover 11% of all the ARG genotypes detected in bacterial genomes.

**Conclusions:**

The above observations indicated that there are both biological and ecological limitations to class 1 integrons in acquiring and spreading ARGs across different classes of the domain *Bacteria*.

**Electronic supplementary material:**

The online version of this article (10.1186/s40168-018-0516-2) contains supplementary material, which is available to authorized users.

## Background

Integrons are the major contributors to the acquisition and dissemination of antibiotic resistance genes (ARGs) [1, 2], and class 1 integrons are the central agents. They have been so successful at spreading into diverse clinically relevant taxa that they have been likened to invasive species [3]. Their subsequent spread into multiple environments has also led to suggestions that the class 1 integron-integrase gene (*intI1*) could be used as a proxy for anthropogenic pollution [4]. Thus, this study mainly focused on the class 1 integrons with typical structure and well-known *intI1*, which are considered to have a significant contribution to the spreading of ARGs.

Class 1 integrons were initially discovered in clinical isolates. However, as more and more metagenomic and qPCR analyses regularly detect *intI1* in different environments [4–8], the comprehensive recovery of integrons in metagenomic data has become a demanding task. The first bottleneck lies in the lack of a well-organized pipeline for integron identification, coupled with a well-curated *intI1* database. Previous studies on class 1 integrons and *intI1* in environments mainly relied on PCR-based sequencing [6, 7, 9, 10], but biases from primers would result in incomplete information. Besides, PCR-independent methods were often designed to target separate integron elements, such as *attC* sites [11] and *intI1* [6], but not whole integrons. Recently, a program targeting whole integrons, the IntegronFinder [12], was designed for complete genomes and does not support draft genomes and metagenomic data.

Without comprehensive databases and methods, it is difficult to evaluate the phylogenetic distribution, ARG content, and the environmental landscape of integrons. Such knowledge is needed to understand their contribution in acquiring and spreading ARGs in environments. To address this goal, we asked two fundamental questions using bacterial whole genomes: (1) what is the potential of class 1 integrons as invasive species across the current bacterial taxa? and (2) what is the contribution of class 1 integrons to the acquisition of antibiotic resistance. Previous studies have revealed that the class 1 integrons carry diverse ARGs and are wildly distributed in more than 70 species [3, 13], especially gram-negative bacterial pathogens. A more comprehensive survey into 2500 complete genomes [12] indicates the heterogeneous distribution of integrons in *Proteobacteria*. This whole genome analysis avoids the bias from PCR primers, while another concern arises of the limited genomes and the exclusion of draft genomes. The NCBI bacterial whole genome database (WGD) and plasmid database [14] have been rapidly expanded to now 73,655 complete and draft (≥ 50% completeness [15, 16] on May 11, 2018) genomes and 12,733 plasmids. Even though these genomes do not represent the uncultured species in the environments, they can serve as representatives of the microbes of interest to microbiologists and clinicians. Bioinformatic analyses based on WGD may provide essential information for interpreting the environments, as demonstrated in recent studies by the construction of database [17, 18] and benchmark references for metagenomic analysis [19–23]. Similarly, the identification of class 1 integrons and their ARGs in WGD would help define their roles in horizontal gene transfer (HGT). Thus, a bioinformatics pipeline was developed to systematically identify class 1 integrons and applied to WGD. The deeper understanding revealed in this study would help future studies to better interpret the functions and potential risks of integrons in environments.

## Results

### Integrase database construction and classification

The previous *intI1* databases [6, 12] were evaluated against all curated class 1 integrons and a subset of potential clinical class 1 integrons from the INTEGRALL [24], which reveals a coverage of less than 30 and 70%, respectively (Additional file 1: Table S1). Thus, the first task was to construct a new *intI1* database to allow a more comprehensive identification of integrons.

**Fig. 1:**
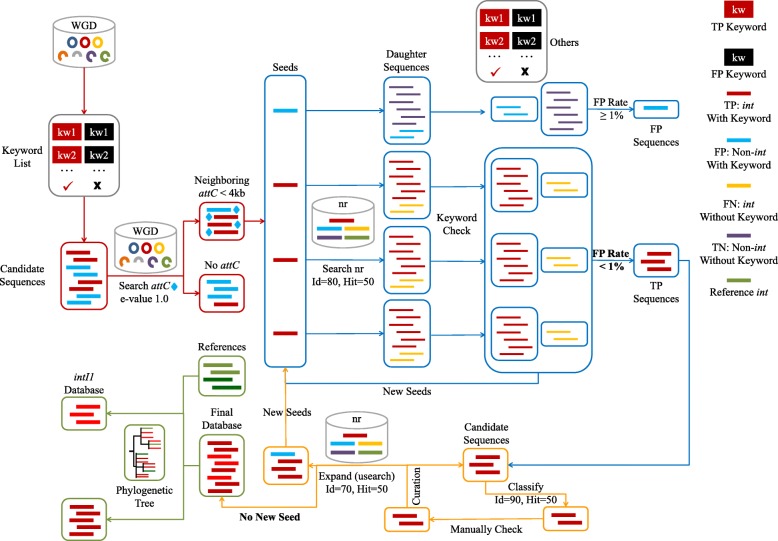
The technical flow of the construction of integron-integrase database. Abbreviations: WGD, whole genome database; Id, (amino acid) identity; Hit, (amino acid) hit length; kw, keyword; TP, true positive; FP, false positive; *int*, integrase genes

A new method is introduced here to construct databases integrating both WGD and NCBI nr database [14] (Fig. 1), by keyword search (Additional file 1: Table S2), sequence-based search, and phylogenetic topology. The WGD collected in this study contains 73,655 complete and draft (≥ 50% completeness on May 11, 2018) genomes (Additional file 1: Table S3). Compared to nr database, WGD has more rigorous and standardized annotations of genes (keyword search) and can provide the information of related or neighboring genes (further filtering). Meanwhile, nr database can provide more abundant sources of both positive- and false-positive sequences for curation and expansion (sequence-based search). Integrases were firstly extracted from WGD by their annotations. After curation and expansion by nr database [14] using BLASTP [25], an integrase database was constructed with 3384 non-redundant sequences (Additional file 1: Figure S1). Together, the previous databases only cover 15% of the integrase database and 70% of the *intI1* database in this study. To avoid false positives caused by misannotation, we removed integrases with no neighboring *attC* site (≤ 4 kb in distance).

**Fig. 2:**
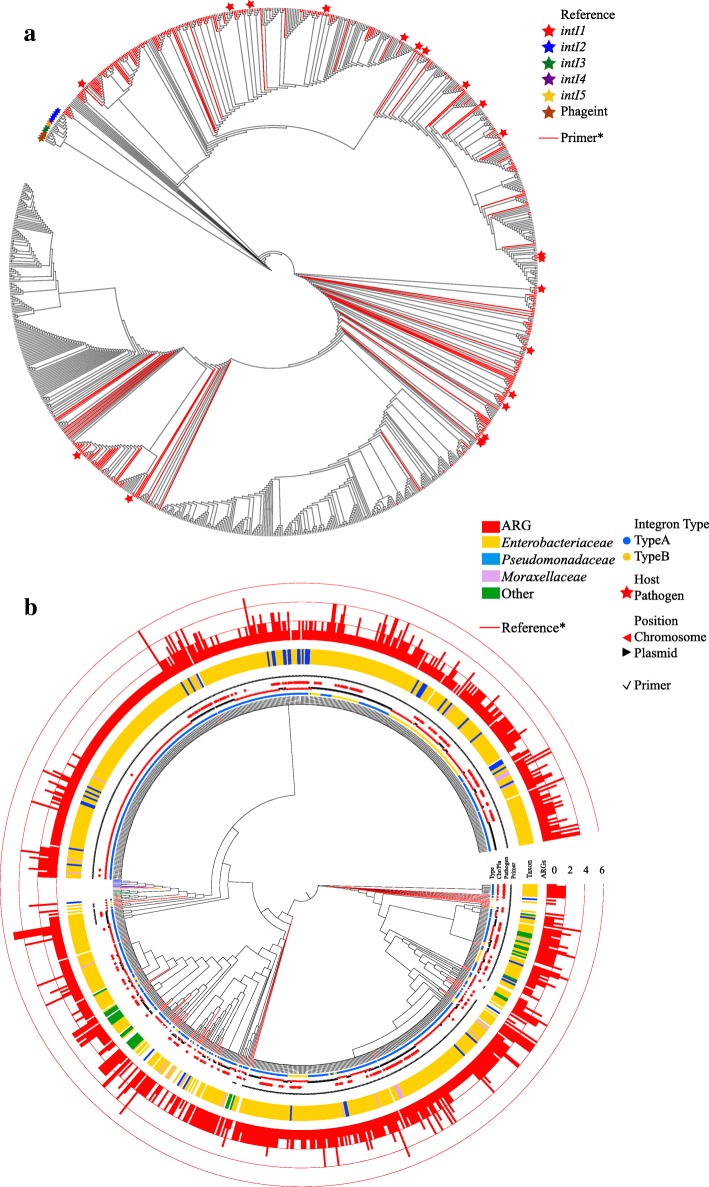
The phylogenetic tree of **a** *intI1* database with the coverage of qPCR primer set [9] (*) and reference information. **b** All extracted *intI1* of types A and B integrons; ARGs carried by the integrons and taxonomic information of the hosts. *The color of the edges representing the reference integrases was consistent the color of stars representing the same references in **a**

The integrases were classified into *intI1* and non-*intI1* by their phylogenetic relationship to references (of classes I to V, *XerC*, and *XerD*) from the NCBI database (Additional file 1: Figure S1). Totally, 922 integrases were identified as *intI1* (Fig. 2a) to populate the *intI1* sub-database and were validated to have both high specificity and coverage. The specificity was evaluated as 100% by searching against WGD using BLASTP [25]. The coverage was evaluated to be 90% (Additional file 1: Table S1) of all clinical class 1 integrons from the INTEGRALL [24].

### Pipeline development

**Fig. 3:**
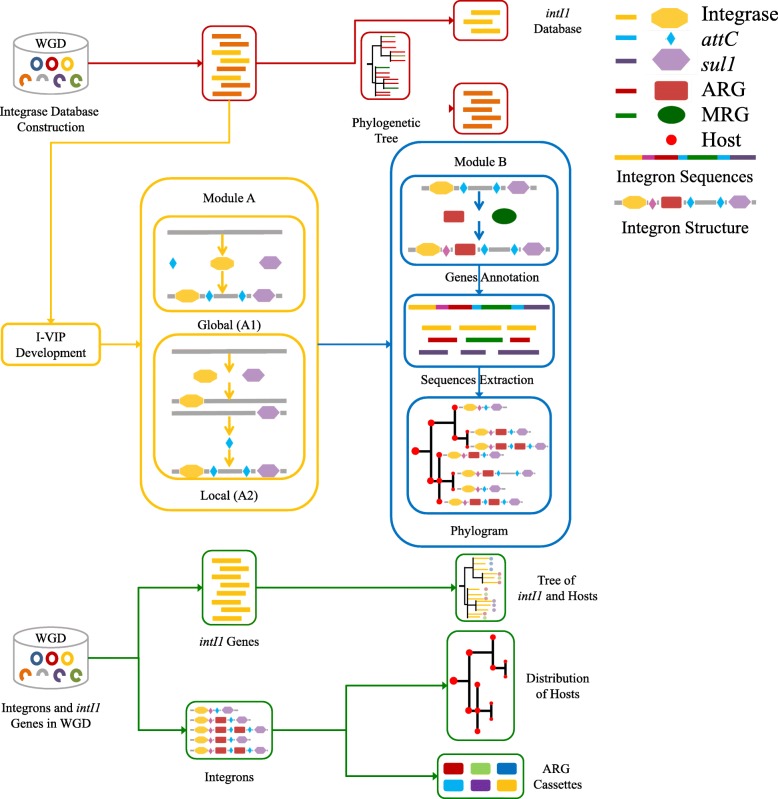
The technical flow of this study. Red framework: integrase database construction. Orange framework: I-VIP module A. Blue framework: I-VIP module B. Green framework: investigation of integrons and *intI1* genes in whole genome database (WGD)

Briefly, the Integron Visualization and Identification Pipeline (I-VIP) is a well-organized pipeline to identify, classify, annotate, and visualize class 1 integrons (Fig. 3 and Additional file 1: Figure S2, Additional file 1: Supplementary Methods) in complete/draft genomes and assembled metagenomes. To facilitate flexible application by the users, I-VIP was separated into two modules: module A for integron identification and classification (orange framework in Fig. 3) and module B for integron extraction, annotation, and visualization (blue framework in Fig. 3).

Firstly, three elements were selected to identify integrons (Additional file 1: Figure S3), which are the *intI1* as the 5′conserved segment (5′-CS), the *attC* site associated with gene cassettes (Gcs), and the sulfonamide resistance gene (*sulI*) as the 3′conserved segment (3′-CS) of class 1 integrons. This identification is conducted using sequence-based searching against three reference databases, the *attC* cite [12], the integrase (this study), and *sulI* [26] databases. Since these databases were constructed by nr database, they included sequences from both cultured and not-yet-cultured bacteria. Thus, the I-VIP is not limited to complete genomes but can also cover integrons from draft genomes and environmental metagenomes. However, a functional integron should be further evaluated by the other integron elements, such as promoters and regulatory genes [1, 27].

In module A, integron elements within a distance of 4 kb (twice the size of the longest Gcs in class 1 integrons [28]) are clustered into one candidate integron. The longest Gc found in WGD was 2.6 kb, indicating that the length criterion of 4 kb for clustering should be sufficient. In this study, an integron is defined as a segment of sequence containing at least two neighboring (≤ 4 kb in distance) integron elements (integrase, *attC* site, or *sulI*) and the genes entrained among these elements. Qualified integrons are then classified into types A to E based on the integron elements they carry (Additional file 1: Figure S3). Integrons with both *intI1* and *sulI* are classified as type A, and integrons with only *intI1* but no *sulI* are classified as type B. Type C integrons refer to integrons carrying integrases of other classes. The classification of types A–C is introduced in this study to differentiate the potential class 1 integrons (types A and B) and integrons of other classes (type C). The class 1 integrons are further separated into types A and B based on the presence of *sulI*. A type A integron has a typical structure of clinical class 1 integrons, while a type B integron could be a potential pre-clinical class 1 integron [27] or could be caused by the deletion of 3′-CS [29]. Since the activity of an integron ultimately hinges on the possession of the functional platform (an integrase and an *attI* site), integrons carrying no integrases are classified into types D (with *sulI* genes) and E (without *sulI* genes) as non-functional integrons. Types D and E integrons are proposed as the sources of Gcs for integrase-carrying integrons (types A to C) or as a means of stabilizing Gcs in genomes [12]. The identification of types D and E integrons at the extremities of assembled contigs should be treated with caution since they could be caused by the inefficient assembly.

For further analysis, the sequences of whole integrons and their integrases and Gcs are extracted using module B. The Gcs are annotated using Structured ARG (SARG) [26] and ComMet [30] databases for potential ARGs and the metal resistant genes (MRGs). The structure of integrons is generated based on the arrangement of integron elements and Gcs and is visualized using the phylogenetic tree of the hosts as the skeleton.

The I-VIP was compared to the IntegronFinder [12] applying compatible settings, using a set of 5436 complete genomes. The classification of *intI1* genes from other integrases and the inclusion of *sulI* as an anchoring element enable the I-VIP to differentiate the potential clinical and pre-clinical class 1 integrons (types A and B) from the integrons of other classes (type C). In contrast, the types A–C integrons are generally identified as complete integrons by IntegronFinder (Additional file 1: Table S4 and Figure S4). Besides, single integrases and single *attC* sites, which are identified as In0 and CALIN with single *attC* by IntegronFinder, are not defined as integrons by I-VIP and are excluded from the results to avoid false positives caused by misannotation. In details, the I-VIP and IntegronFinder recovered 1320 and 920 integrons (not considering the single integrases and single *attC* cites), respectively, from the set of complete genomes. Totally, 845 integrons (153 type A, 38 type B, 154 type C, 34 type D, and 466 type E) are shared by two approaches, with minor differences in the number of Gcs. However, the *attC* sites of 34 type A–C integrons are missed by IntegronFinder and therefore classified as In0. Moreover, the difference of the integrase databases used by two approaches resulted in the missing of the integrases on 294 integrons by IntegronFinder compared to 99 missing integrases by I-VIP. The integrase database in this study also recovered 69 type C integrons, which were not detected by the IntegronFinder.

### Integrons in WGD and plasmid database

Because of the uniqueness and dynamics of bacterial genomes [15, 31], the inclusion of draft genomes would largely expand the diversity of genome reservoirs, especially for mobile gene elements (MGEs) like integrons. Thus, the I-VIP was designed to be applicable to both complete and draft genomes in the current bacterial WGD. To further assess the potential for HGT, especially by conjugative transformation [32], all sequences in WGD were classified into chromosomes and plasmids according to the GenBank annotation and were curated by PlasFlow [33] (Additional file 1: Table S3). As a supplement to the plasmids retrieved from WGD, the NCBI plasmid database containing totally 12,733 plasmids was downloaded and analyzed by I-VIP. Although some MGEs, such as transposons, would facilitate the transfer of chromosomal integrons, the identification of such elements and the evaluation of their mobility are not the focus of this study.

Totally, 2977 integrons were identified from the WGD and plasmid database (Table 1 and Additional file 1: Table S5). The integrons were classified into types A to E based on their integron elements (Table 1) and more than two thirds of the integrons (2137) carry the integrases (types A–C). The class 1 integrons account for half of all the identified integrons, consisting of 1039 clinical class 1 integrons (type A) and 431 pre-clinical class 1 integrons (type B). Approximately 50% of the class 1 integrons (types A and B) are carried by plasmids, showing a potential of HGT by conjugative transformation; while more than 90% of the integrons of other classes (type C) are embedded in chromosomes.

**Table 1:** Summary of the integron distribution in the collection of 73,655 all currently available bacteria whole (complete and draft) genomes identified by I-VIP developed in this study. All sequences in bacteria whole (complete and draft) genomes were annotated into chromosomes and plasmids by NCBI GenBank annotation and curated by PlasFlow. The chromosomes/plasmids of complete genomes annotated by PlasFlow were compared to the GenBank annotation, as indicated in the brackets

Database	Position	Integron types	Total No.	A	B	C	D	E
WGD complete	Chromosome	All integrons	1182	106 (97)	40 (35)	490 (486)	24 (22)	522 (455)
		ARG-carrying integrons	215	103	37	36	13	26
	Plasmid	All integrons	403	232 (241)	83 (88)	0 (4)	21 (23)	67 (134)
		ARG-carrying integrons	371	229	81	0	20	41
WGD draft	Chromosome	All integrons	849	419	221	111	67	31
		ARG-carrying integrons	716	412	216	11	41	36
	Plasmid	All integrons	6	2	3	0	0	1
		ARG-carrying integrons	7	2	3	0	0	2
Plasmids	Plasmid	All integrons	537	280	84	60	18	95
		ARG-carrying integrons	478	278	79	54	18	49

### Phylogenetic distribution of integrons in WGD

Among those 2440 integrons identified in WGD, 2295 integrons are hosted by the phylum *Proteobacteria* (30,593 genomes), mainly by *Gammaproteobacteria* (93%) (Additional file 1: Table S6). In contrast, the phyla *Tenericutes* (392 genomes) and *Chlamydiae* (336 genomes) encode no integron. This is not surprising because *Tenericutes* and *Chlamydiae* are known to have limited capacity for HGT. Moreover, it is intriguing to find a heterogeneous distribution of integrons in phylogenetically close clades under the same taxonomic level. For example, there are only a few integrons detected in *Alphaproteobacteria* (5 in 2879 available genomes) and *Betaproteobacteria* (109 in 3401 genomes) while their sister class *Gammaproteobacteria* harbors 2142 integrons (among 21,986 genomes) in total, in agreement with a previous study into 2500 complete genomes [12].

**Fig. 4:**
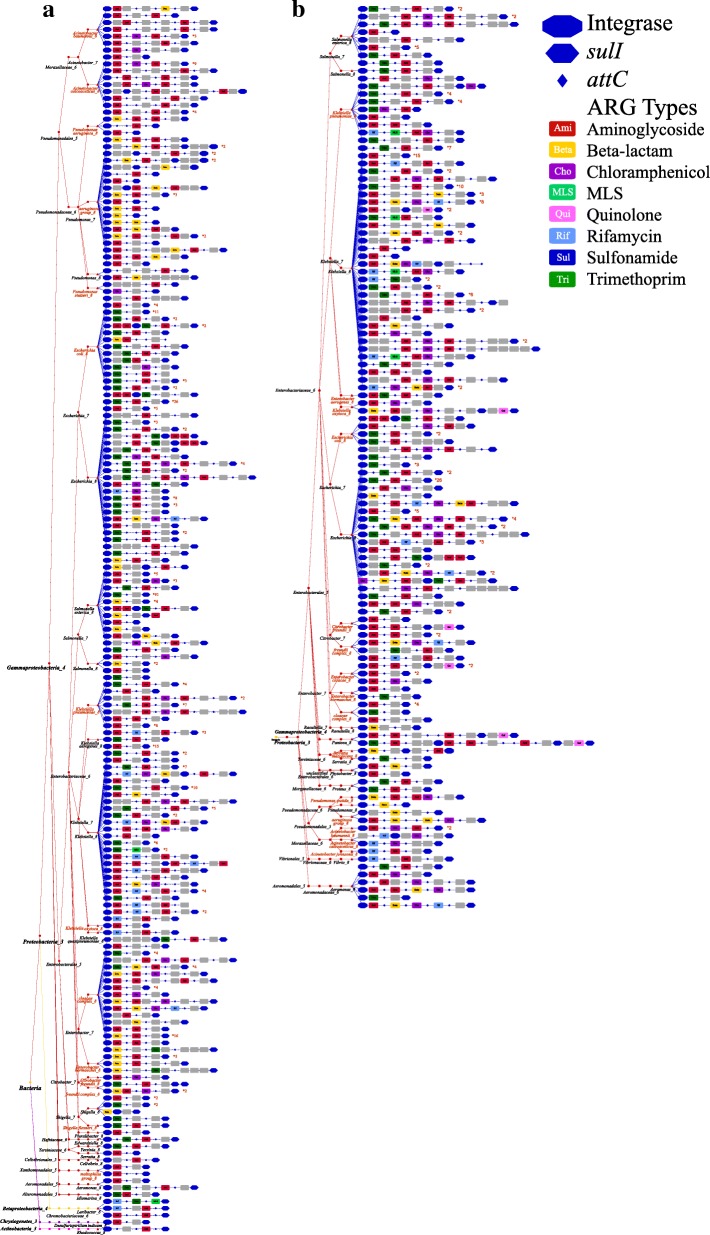
The phylogram of type A integrons carrying ARGs normalized against the species level, on **a** chromosomes and **b** plasmids. The types of integron elements were differentiated by symbols. The structure of integrons was merged into a phylogenetic tree of their hosts to construct the phylogram

To avoid the bias caused by heterogeneous sequencing and pseudo-replication of draft genomes, especially in pathogenic species, all genomes within a species were integrated to count the occurrence of integrons. The integron copy number was normalized against the species level, that is, one copy integron per species was counted if any one of the genomes within that species is carrying an integron (Additional file 1: Table S7). For type A (clinical class 1) integrons, it demonstrates a clear trend that they are highly dominant in pathogenic hosts and frequently carried ARGs, which indicate their primary roles in HGT of ARGs (Fig. 4). Firstly, the host species of type A integrons reveals a significant pathogenic prevalence of 58%, which means that 58% of the bacterial species harboring type A integrons are potential human pathogens. It is almost seven times compared to the percentage of the pathogenic species in WGD (Table 2). Although the hosts of type A integrons are dominant in class *Gammaproteobacteria* and family *Enterobacteriaceae* with high percentage of pathogenic species (14 and 25%), the significantly higher pathogenic prevalence of type A integrons (60 and 54%) clearly implies the ecological adaptations of clinical class 1 integrons in bacteria of medical importance. In addition, more than half of type A integrons are chromosomal integrons (Table 1) showing less mobility of HGT by conjugative transformation. These observations may indicate that so far, the HGT of clinical class 1 integrons could be restricted even within *Gammaproteobacteria*. Although the current WGD is biased to more easily cultured and pathogenic strains, and the HGT of class 1 integrons is variable to the bacterial communities and the environments, the very strong trend is suggestive that there could be a biological [27] barrier (i.e., mechanistic gene transfer [32, 34, 35]) and ecological barrier (i.e., host fitness effects [36]) to broad HGT of class 1 integrons. Furthermore, almost all (99%) type A integrons carry at least one ARG, mainly harbored by the pathogenic species such as *Acinetobacter baumannii*, *Pseudomonas aeruginosa*, *Shigella flexneri*, *Klebsiella oxytoca*, *Klebsiella pneumoniae*, *Enterobacter* species, and *Escherichia coli* (of *Proteobacteria*), covering four of the species named “ESKAPE” pathogens [37]. In contrast, the other two species in the “ESKAPE” group, the *Enterococcus faecium* and *Staphylococcus aureus* (of phylum *Firmicutes*), carry only four types D and E integrons (without ARG). It suggests that for human pathogenic species, the biological barrier (across phyla) could have played a greater role than the ecological barrier as the limitation for HGT of clinical class 1 integrons.

**Table 2:** Distribution of type A integrons and the 73,655 bacterial genomes in different phyla, classes, families, and genera. The integron copy number was normalized against the species level

Phylum/class/family/genus	No. of species	No. of species carrying type A integrons	Pre%A^a^	Pre%Total^b^
*Bacteria*	4310	52	57.7%	8.6%
*Proteobacteria*	1831	50	58.0%	9.8%
*Gammaproteobacteria*	832	48	60.4%	14.2%
*Enterobacteriaceae*	102	24	54.2%	25.5%
*Klebsiella*	9	7	57.1%	44.4%
*Enterobacter*	10	4	75.0%	40.0%
*Escherichia*	6	4	50.0%	33.3%
Other genera in *Enterobacteriaceae*	77	9	44.4%	33.7%
*Pseudomonadaceae*	49	7	71.4%	14.3%
*Pseudomonas*	44	7	71.4%	15.9%
*Moraxellaceae*	44	3	100.0%	22.7%
*Acinetobacter*	31	3	100.0%	25.8%
Other families in *Gammaproteobacteria*	637	14	57.4%	18.5%
*Betaproteobacteria*	318	2	0.0%	10.4%
*Actinobacteria*	441	1	100.0%	10.9%
*Chrysiogenetes*	3	1	0.0%	0.0%

^a^The prevalence of host species carrying type A integrons = no. of pathogenic species carrying type A integrons/total no. of species carrying type A integrons

^b^The prevalence of all species = no. of pathogenic species/total no. of species

In WGD, the majority (90%) of the hosts carry one copy of type A integrons on either chromosomes or plasmids (Additional file 1: Figure S5). It is intriguing that eight hosts from the genus of *Klebsiella* and *Salmonella* carry both chromosomal and plasmid type A integrons. It is not surprising that 88% of the plasmid type A integrons in WGD are hosted by pathogenic species (Additional file 1: Table S5), as they were originally isolated from clinical settings. The non-pathogenic species harboring the remaining 12% type A plasmid integrons are highly dominated by the close relatives of pathogens (especially in the family of *Enterobacteriaceae*). These integrons all carry ARGs and could serve as an important reservoir for HGT of ARGs into human pathogens [2], especially since they occupy the same habitats.

### ARGs on class 1 integrons

**Fig. 5:**
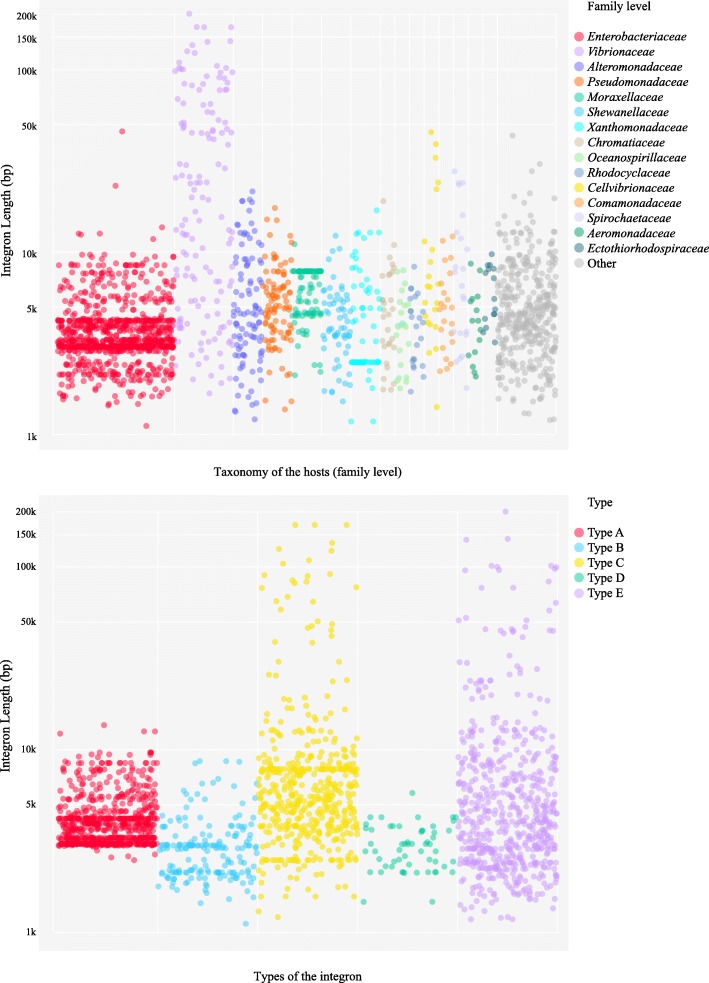
Size (log 2 value of total length) distribution of all 2977 chromosomal and plasmid integrons. Integrons were classified based on the taxonomic information of their hosts (family level) and the types of A to E

Types A and B integrons generally carry 0 to 11 Gcs, with a total size of 1 to 14 kb (from 3′-CS to 5′-CS, Fig. 5). The majority (99%) of type A integrons have the size of 3 to 10 kb. Overall, most (81%) of the ARG-carrying integrons are type A and type B integrons (class 1 integrons), pointing out that the class 1 integrons play the central role in the dissemination and the acquisition of ARGs, compared to the integrons of other classes. Specifically, almost all (98%) class 1 integrons carry at least one ARG, and more than half of them carry multiple (2–6) ARGs. Moreover, 48% of the Gcs on class 1 integrons are annotated with the function of antibiotic resistance. Although ARGs have been frequently captured by class 1 integrons, it reveals a limited diversity and heterogeneous distribution of totally 60 ARG genotypes and 9 ARG phenotypes, compared to a total of 1209 genotypes and 24 phenotypes in SARG database [26]. ARGs resistant to aminoglycoside, beta-lactam, chloramphenicol, and trimethoprim are widely distributed in both chromosomal and plasmid class 1 integrons (Fig. 4 and Additional file 1: Table S8). In contrast, macrolide-lincosamide-streptogramin (MLS)-, quinolone-, and tetracycline-resistant genes were rarely detected on the class 1 integrons. Regardless of the high occurrence of tetracycline-resistant genes in bacterial genomes and in the environments [30, 38], only one tetracycline resistance protein was discovered to be acquired by a plasmid type B integron. Besides, the plasmid class 1 integrons harbor a more diverse reservoir of ARGs (59 genotypes) than the chromosomal class 1 integrons (45 genotypes), including the medically relevant ARGs of *TEM-1* and *VIM-1* resistant to beta-lactam [39].

### The phylogenetic conservation of *intI1*

To identify the host of *intI1*, the *intI1* sequences of class 1 integrons in WGD were extracted and further classified into two subgroups, i.e., (1) the type A *intI1* carried by the type A integrons and (2) the type B *intI1* carried by the type B integrons. The amino acid sequence identity in the type A *intI1* varies from 56 to 100%, indicating that these clinical class 1 integrons may not originate from one common ancestor [2]. The mutational inactivation to be adapted to different hosts could have exaggerated the divergence within the type A *intI1* genes [36]. Figure 2b displays the phylogenetic relationship among 693 type A *intI1* genes and type B 155 *intI1* genes, which demonstrates that (1) the *intI1* is harbored mostly (85%) by the *Enterobacteriaceae* family in *Gammaproteobacteria* and (2) almost all (96%) *intI1* is embedded in ARG-carrying integrons and more than half (53%) of *intI1* is leading multiple ARGs. It is intriguing that a large portion (74% on Fig. 2b) of *intI1* shows no phylogenetic difference, distributed in both pathogenic and non-pathogenic hosts in *Enterobacteriaceae*, *Pseudomonadaceae*, *Moraxellaceae*, and other families, indicating the potential for HGT of these *intI1* across the family level.

### Database summary

In this study, two databases (integron and integrase) were rigorously constructed and curated using the bioinformatic pipeline and whole genome analysis, which overcome the inherent limitation and biases of databases constructed from PCR-generated sequences. With the rapid expansion of available genomes, periodic updates of these databases could be performed through the automatic pipeline developed in this study, enabling a comprehensive survey on integrons.

The integrase database (Additional file 1: Supplementary File S1) contained 3384 complete and non-redundant integrases with an aa inter-group identity of 17–100%. A total of 922 integrases were classified as *intI1* to generate a sub-database, the *intI1* database. The low coverage (26%) of previous *intI1* primers [9] (HS464/HS463a) against the corresponding *intI1* nucleotide (nt) database in this study indicates that the currently available primer [9] may only identify a limited spectrum (Fig. 2a red edges, Additional file 1: Table S9) of the *intI1* database. Thus, new primer sets will be necessary for a more comprehensive identification and quantification of *intI1* from clinical and environmental samples.

The integron database is a compact database of both sequences and associated information for 2153 non-redundant whole integrons (Additional file 1: Table S5), 66% of which have not been included by the INTEGRALL [24] (95% aa identity and 90% hit length, Additional file 1: Table S10). A total of 502 type A and 283 type B non-redundant integrons dramatically expand the diversity of class 1 integrons in the INTEGRALL database with an addition of 61% new sequences. However, the INTEGRALL database also contributes new integron sequences with a variation of gene cassettes (but not new integrases), and the integration of these two databases would provide the abundant and representative resources for future studies. Moreover, the available PCR primer sets [5, 40–42] have very low coverages of 0 to 24% (Additional file 1: Table S9) of the integron database in this study, indicating that a large proportion of integrons in the environments may be missed using those primer sets.

## Discussion and conclusions

By exploring the integrons from a WGD of 73,655 complete and draft bacterial genomes and a plasmid database of 12,733 plasmids using the I-VIP pipeline developed in this study, the contribution of class 1 integrons was evaluated to spread and acquire ARGs.

Firstly, the host species of class 1 integrons are found to be highly conserved within one narrow bacterial lineage in *Gammaproteobacteria*, especially in three families of *Enterobacteriaceae*, *Pseudomonadaceae*, and *Moraxellaceae*. By extracting the metadata of their hosts from the IMG database, the habitat information was recovered for 20% of the class 1 integrons, which revealed that most of these 20% class 1 integrons distributed in human-associated habitats. The significantly high prevalence of clinical class 1 integrons in potential human pathogens was observed (with sevenfold of the percentage of pathogenic bacterial species in WGD), but they are also commonly distributed (the remaining 42%) in closely related non-pathogenic *Gammaproteobacteria*. Moreover, more than half of the class 1 integrons are embedded in chromosomes, which indicates less potential of HGT by conjugative transformation. This strongly suggests that the contribution of class 1 integrons on the ARG dissemination might be limited, in terms of the biological potential of HGT (mainly within *Gammaproteobacteria*), but critical, in terms of exchanging Gcs between non-pathogens and pathogens.

The role of integrons is well established in supplying adaptive traits via HGT to bacteria under various selection pressures [2, 3]. However, this does not mean that integrons harbor resistance to all antimicrobial agents. In fact, after exploring the ARGs already acquired by the integrons on chromosomes and plasmids, the ARGs harbored on all types of integrons are surprisingly limited to only 69 genotypes from 9 phenotypes of ARGs (Additional file 1: Table S8), i.e., the aminoglycosides, sulfonamide, trimethoprim, beta-lactam, chloramphenicol, and rifamycin. Among 11 phenotypes of globally used antibiotics [43], the resistant genes to tetracycline, vancomycin, and polymyxin and multidrug-resistant genes are rarely captured on the integrons. In contrast, ARGs from 627 genotypes and 24 phenotypes are found to be widely distributed across the bacterial taxa in WGD. It seemed that 89% of the ARG genotypes in bacterial genomes are not likely to be acquired and expressed via the integrons. This result is consistent with a previous environmental survey that reported limited genotypes of ARGs carried by class 1 integrons [6]. These observations suggest that high co-occurrence of *intI1* and ARGs of other genotypes [4, 44] could be caused by the co-enrichment and co-localization of class 1 integrons and these ARGs on the same transposons or plasmids. Although it deserves confirmation in the further studies, the datasets of WGD and plasmid database used in this study could serve as appropriate representatives for natural environments.

The above two observations, which are (1) the potential of class 1 integrons as invasive species is constrained within *Gammaproteobacteria* and (2) the contribution of class 1 integrons to the acquisition of antibiotic resistance is limited to 69 genotypes (11% of the ARG genotypes identified in WGD), indicate that there are both biological and ecological limitations to class 1 integrons in acquiring and spreading ARGs across different classes of the domain *Bacteria* by HGT.

The complete genomes used in this study are mainly obtained from culturable species of practical importance to humans. To investigate the environmental microbes, especially the not-yet-cultured species, all the draft genomes from NCBI genome database, including 7351 bacterial genomes obtained in a previous study from environmental and non-human gastrointestinal samples [15], were downloaded and analyzed by I-VIP. By recovering the metadata of 10,733 genomes (Additional file 1: Table S3) from the columns “Cultured,” “Ecosystem,” and “Ecosystem Subtype” in IMG database, the proportion of not-yet-cultured genomes was evaluated to be 26% in WGD, which were obtained from 36 diverse environmental and engineered habitats. Although these genomes represent bacterial phylogeny quite well (34 phyla excluding unclassified and *Candidatus* phyla), they do not represent the diversity of the very large not-yet-cultured species in nature. Moreover, the limitation of assembly methods may result in more incomplete integrons (types B, D, and E). To maintain the high quality of contigs, draft genomes with less than 50% completeness [15, 16] were abandoned. Additionally, the impact of short-read assembly on the recovery of complete integrons was assessed by comparing the proportion of types B, D, and E integrons obtained from the complete genomes against the draft genomes within the same family of *Enterobacteriaceae*. The results show no significant enrichment of the types B, D, and E integrons in the draft genomes (40% in complete genomes compared with 42% in draft genomes). Furthermore, the development of long-read sequencing, i.e., PacBio [45] and MinION nanopore [46], and of assembly-facilitated technologies, i.e., inverse PCR [47] and Hi-C [48, 49], will help obtain draft or nearly complete genomes with high quality, as demonstrated by previous studies into the pure cultures [50, 51]. In addition, the I-VIP pipeline can rapidly take advantage of the expanding WGD to broaden the bacterial taxa examined and incorporate new metagenomic datasets. These data will be crucial for understanding the distribution and dissemination of integrons under the continuing influence of anthropogenic impacts. Besides, the approach used in this study focuses on the classical class 1 integrons that are already known and well investigated. But it is probably just a small part of the class 1 integron distribution and will not reflect its biology to a full extent. Moreover, it is challenging to unravel the complex resistance regions computationally.

## Methods

The overall technical flow of this study is summarized in Fig. 3.

### Bacterial WGD

This database contains a collection of 73,655 bacterial genomes (10,112 complete genomes and 63,543 draft genomes with ≥ 50% completeness [15, 16]) and 12,733 plasmids (ftp://ftp.ncbi.nlm.nih.gov/genomes/genbank/bacteria) and NCBI plasmid database (ftp://ftp.ncbi.nlm.nih.gov/genomes/refseq/plasmid) [14] (on May 11, 2018). The taxonomy information of all genomes was curated and summarized in Additional file 1: Table S3. The potential pathogenicity of bacterial genomes was obtained by matching their taxonomic information with a database of all recognized species of human bacterial pathogens [52]. If either one of the taxonomic annotations of species or strain level was matched to the human pathogen list [52], the genome was labeled as a potential human pathogen [30, 53]. Those genomes without the taxonomic annotations down to the genus level were treated as non-pathogens. The information of habitats and isolates was extracted from IMG database (on May 10, 2018) combining the columns of “*Cultured*,” “*Ecosystem*,” “*Ecosystem Category*,” “*Ecosystem Subtype*,” “*Ecosystem Type*,” and “*Relevance*.” By mapping the organism name of genomes, the metadata of 10,733 genomes was recovered with valid information of the columns “Cultured,” “Ecosystem,” and “Ecosystem Subtype” (Additional file 1: Table S3). All sequences/contigs with the size larger than 1 kb in WGD (both the complete and draft genomes) were classified into chromosomes and plasmids by the PlasFlow 1.0 [33] using the default threshold of 0.7 for probability filtering.

### Integrase database construction

A new method was developed here to construct databases by integrating WGD and NCBI nr database [14] (Fig. 1, Additional file 1: Supplementary Methods), which could facilitate future studies to construct databases of other functional genes. Candidate integrases (containing true-positive keywords and no false-positive keyword) were firstly extracted from WGD by keyword search (Additional file 1: Table S2) against their annotation (red framework in Fig. 1). Then, sequences containing integrases were searched for *attC* site [12] (*e* value 1) by cmsearch 1.1.1 [54], and candidate integrases neighboring *attC* sites (≤ 4 kb in distance) were kept as seed sequences for curation by nr database (blue framework in Fig. 1). The seed sequences were sequentially searched against nr database by BLASTP 2.2.28+ [25] (*e* value 1e−3, 80% aa similarity over 50% aa hit length), and the output sequences from nr database were treated as daughter sequences. The annotation of the daughter sequences was used to evaluate their seed sequences by calculating the ratio of false-positive daughter sequences. In other words, those seed sequences were abandoned if larger than 1% of their daughter sequences contained false-positive keywords or did not contain any true-positive keyword. All the daughter sequences of true-positive seed sequences were treated as new seed sequences for the next cycle of curation by nr database. After curation, all true-positive sequences were classified into subgroups, and one representative for each subgroup was manually checked by online BLASTP [25] (orange framework in Fig. 1). Those subgroups with false-positive representative were abandoned. After manually checking, the candidate database was expanded against nr database using usearchv8 [55] (*e* value 1e−3, 70% aa similarity over 50% aa hit length). All daughter sequences were treated as new seed sequences and were curated one by one using the same method described before (blue framework in Fig. 1). The cycles of curation and expansion (blue and orange framework in Fig. 1) were ended if no new seed sequence was found. Finally, the *intI1* genes were classified by the phylogenetic tree, using default parameters of MUSCLE v3.8.31 [56] and FastTree 2.1.10 [57] (green framework in Fig. 1).

The coverage of the integrase databases constructed in previous studies and this study was evaluated against all curated class 1 integrons from INTEGRALL [24] (http://integrall.bio.ua.pt/IntegronNumbering-lastestUpdate.xls), while still it could be heavily biased to mobile integrons with ARGs. The cutoff for different databases was consistent with the original studies (*e* value 1e−5, 90% nt similarity over 50% nt hit length for [6]; *e* value 1e−3 over 50% aa hit length for [12]) and this study (*e* value 1e−3, 80% aa similarity over 50% aa hit length). The in silico coverage of previously designed primers to *intI1* nucleotide (nt) database and the class 1 integron database was evaluated by self-written scripts and PRISE2 [58], allowing no mismatch.

### Integron identification and visualization

The integrons were identified, classified, annotated, and visualized by I-VIP (Fig. 3 and Additional file 1: Figure S2, Additional file 1: Supplementary Methods), allowing multiple inputs (available on https://github.com/caozhichongchong/I-VIP/releases). The identification was conducted against three reference databases, by cmsearch 1.1.1 [54] against the *attC* database [12] (*e* value 1 for 2 Mb), by BLASTP 2.2.28+ [25] against the integrase database (this study, *e* value 1e−3, 80% aa similarity over 50% aa hit length), and *sulI* database [26] (*e* value 1e−3, 90% aa similarity over 80% aa hit length). To maintain the consistency of the *e* value for cmsearch, all genomes were split into 2 Mb. In this study, to avoid potential misidentification by similarity search, integrons with only one integron element (integrase, *attC* cite, or *sulI*) were excluded. The Gcs on integrons were extracted and annotated by BLASTP 2.2.28+ [25] against SARG [26] (*e* value 1e−5, 90% aa similarity over 80% aa hit length) and ComMet database [30] (*e* value 1e−5, 80% aa similarity over 90% aa hit length). Finally, combining the taxonomic information of their hosts, the phylogenetic distribution and the structure of integrons were plotted together as an integron phylogram by *Hierarchic* or *Tree* layout in Cytoscape 3.3.0 [59], using the phylogenetic tree of the hosts as the skeleton.

Since global searches for *attC* sites by cmsearch (module A1) were computationally time-consuming, an alternative process (local search module A2) was developed and compared with module A1. Briefly, module A1 searched the whole profile of the input file for all three integron elements (*attC*, integrase, and *sulI*), while module A2 firstly searched the integrase and then searched the *attC* sites and *sulI* on integrase-containing sequences. Hence, integrons without integrases (types D and E) would not be identified by module A2. However, types A to C integrons were retrieved with 100% specificity and 100% coverage as by module A1 while saving 96% of computation time (evaluated by 10,112 complete genomes). Module A2 could dramatically save time and resources for cmsearch, without sacrificing the accuracy and coverage. The integrons in a set of 5436 complete genomes were identified by IntegronFinder v1.4 [12] applying default settings.

## Additional file

Additional file 1:  The integron-integrase database constructed and curated using bioinformatics pipeline and whole genome analysis in this study, covering 3,384 complete and non-redundant integrases. (ZIP 11490 kb)
