# Supplementary material for: Conserved phylogenetic distribution and limited antibiotic resistance of class 1 integrons revealed by assessing the bacterial genome and plasmid collection
Source: Microbiome. 2018 Jul 21;6:130. doi: 10.1186/s40168-018-0516-2 (PMC6054849; doi:10.1186/s40168-018-0516-2)
Supplement: Supplementary file 1 — The integron-integrase database constructed and curated using bioinformatics pipeline and whole genome analysis in this study, covering 3,384 complete and non-redundant integrases. (ZIP 11490 kb) [file 40168_2018_516_MOESM1_ESM.zip › Supplementary_Information.docx]

**Supplementary Information**

**Title:** **Conserved Phylogenetic Distribution and Limited Antibiotic Resistance of Class 1 Integrons Revealed by Assessing the Bacterial Genome and Plasmid Collection**

An Ni Zhang^1^, Li-Guan Li^1^, Liping Ma^1^, Michael R Gillings^2^, James M Tiedje^3^, Tong Zhang^1,4^*

^1^Environmental Biotechnology Laboratory, The University of Hong Kong, Hong Kong;

^2^Department of Biological Sciences, Species Spectrum Research Centre, Macquarie University, Sydney, New South Wales, Australia;

^3^Center for Microbial Ecology, Michigan State University, East Lansing, MI, USA;

^4^International Center for Antibiotics and Resistance in Environments, Southern University of Science and Technology, China.

* To whom correspondence should be addressed. Tel: +852-2857 8551; Fax: +852-2859 8987; Email: [zhangt@hku.hk](mailto:zhangt@hku.hk) and/or [zhangt3@sustc.edu.cn](mailto:zhangt3@sustc.edu.cn)

**Supplementary Methods**

**Integron Visualization and Identification Pipeline (I-VIP)**

I-VIP consists of two consecutive Modules, Module A for integron identification and Module B for integron sequences extraction, annotation and structure analysis (Fig 3, Supplementary Methods).

To identify integron in both complete and draft bacterial genomes and assembled metagenomes, three composite elements (integron-integrase, *attC* site and *sulI*), were individually searched by sequence-based tools against the *attC* site and *sulI* databases constructed in previous studies[1, 2] and the integron-integrase (referred to as integrase) database in this study described in Method. Integron elements with loci as close as 4 kb[1, 3] were merged into one cluster as one potential integron. In this study, an integron was defined as a segment of sequence covering a cluster of at least two integron elements (containing at least one *attC*) to avoid miss-identification caused by mismatch. Integrons were further classified into A to E Types by the elements they harbored, specifically by the existence of integrase and *sulI* elements (Fig S3). Since the *attC* search by cmsearch 1.1.1[4] (using –max/mid option) was the most time-consuming step of I-VIP, two alternative processes, global search Module A1 and local search Module A2, were provided for users. In Module A1, *attC* site is searched in the whole data input simultaneously with the integrase and *sulI* search; while in Module A2, integrase was firstly searched in the whole data to extract integron-like sequences (segments of sequences containing integrases) and only these integron-like sequences were further searched for *attC* site and *sulI*. This pre-filtering step dramatically decreases the time consumption by 24 times evaluated based on the collection of 7,770 bacteria complete genomes. However, only integrons with integrases will be identified (Types A and B) using Module A2 local search.

By Module B, gene cassettes and sequences of integrons were extracted according to the termini loci of integrons identified in Module A; meanwhile, the elements arrangement (structure) of integrons was constructed in the output files. The integron structure and its gene cassettes was visualized by *Hierarchic* or *Tree* layout in Cytoscape[5], which clearly differentiates the Types of integron elements by shape and color. Furthermore, an integron phylogram can be constructed by merging the integron structure into a phylogenetic tree of their hosts, when the taxonomic information of the hosts was provided. If annotation databases, such as antibiotic resistant gene (ARG) and metal resistant gene (MRG) databases[2, 6], were provided, gene cassettes were further annotated and combined into the structure list and the integron phylogram.

**Integrase database construction and curation**

A new method was developed here to construct databases by integrating the bacterial genome collection and the NCBI nr database[7] (Fig 1), which could facility future studies to construct and expand databases of other functional genes. Compared to nr database, the bacterial genome collection had more rigorous and standardized features/annotations of the target genes (keyword search) and can provide the information of related or neighboring genes (further filtering). Meanwhile, the nr database can provide more abundant sources of both positive and false positive sequences for curation and expansion (sequence-based search). Candidate integrase sequences (containing only the true positive keywords and no false positive keyword) were firstly extracted from all genes of the genome collection by keyword search (Table S2) against their annotation/feature (red framework in Fig 1). The false positive keyword lists contained possible annotation of inactivated integrase due to genetic changes in the ORFs. Then, the sequences containing the candidate integrases were searched for *attC* site[1] (e-value 1 for 2 Mb) by cmsearch 1.1.1[4] and candidates neighboring *attC* sites (no more than 4 kb in distance) were kept as seed sequences for curation by NCBI nr database (blue framework in Fig 1). The seed sequences were sequentially searched against the nr database by BLASTP 2.2.28+[8] (e-value 1e-3, 80% aa similarity over 50% aa hit length) and the output sequences from nr database were treated as daughter sequences. The annotation of the daughter sequences was extracted to evaluate the seed sequences by calculating the false positive rate of daughter sequences. In other words, those seed sequences were abandoned if larger than 1% of their daughter sequences contained false positive keywords or didn’t contain any true positive keyword (false positive sequences). All the daughter sequences (true positive or false positive) of true positive seed sequences were treated as new seed sequences for the next cycle of curation by nr database. After curation, all true positive sequences were firstly classified by BLASTP 2.2.28+[8] (e-value 1e-3, 90% aa similarity over 50% aa hit length) into sub-groups (orange framework in Fig 1). One representative sequence for each sub-group was manually checked by online BLASTP[8] against nr database according to the top ten best hits. Those sub-groups with false positive representativeness were abandoned. After manually checking, the candidate database was expanded against nr database (orange framework in Fig 1) using usearchv8[9] (e-value 1e-3, 70% aa similarity over 50% aa hit length). All the daughter sequences of the candidate database were treated as new seed sequences and were curated one by one using the same method described before (blue framework in Fig 1). The cycles of curation and expansion (blue and orange framework in Fig 1) were ended if no new seed sequence was found. Finally, the *intI1* sequences were classified by the phylogenetic tree, using default parameters of MUSCLE v3.8.31[10] and FastTree 2.1.10[11], in comparison to the reference sequences[7] (green framework in Fig 1). The *intI1* sub-database was constructed by all the *intI1* sequences.

The coverage of the integrase databases constructed in previous studies and this study were evaluated against all 1,011 proposed complete class 1 integron sequences and a subset of 256 clinical class 1 integrons (with *sulI*) from the INTEGRALL database[12]. Here only the unique and complete class I integrons curated by INTEGRALL were used (<http://integrall.bio.ua.pt/IntegronNumbering-lastestUpdate.xls>), still it could be heavily biased towards mobile integrons with ARGs. The cutoff for different databases was consistent with the original studies (e-value 1e-5, 90% nt similarity over 50% nt hit length for *intI1* database[13]; e-value 1e-3 over 50% aa hit length for *intI*_Cterm database[1]) and with this study (e-value 1e-3, 80% aa similarity over 50% aa hit length). The *in silico* coverage of previously designed primers to the *intI1* nucleotide (nt) database and the class 1 integron database was evaluated by self-written scripts and PRISE2[14], allowing no mismatch (100% identity over 100% hit length).

Specificity of *intI1* sequences was further validated using the daughter sequences obtained from the bacterial genome collection by similarity search by BLASTP 2.2.28+[8] with the same cutoff (e-value 1e-3, 80% aa similarity over 50% aa hit length). The newly obtained daughter sequences and annotations were extracted from GenBank files to calculate the false positive rate using the same criteria for the database curation. Also the sequences containing the daughter sequences were searched for *attC* site[1] (e-value 1 for 2 Mb) by cmsearch 1.1.1[4] to check their neighboring *attC* sites.

**Supplementary Figures and Tables**


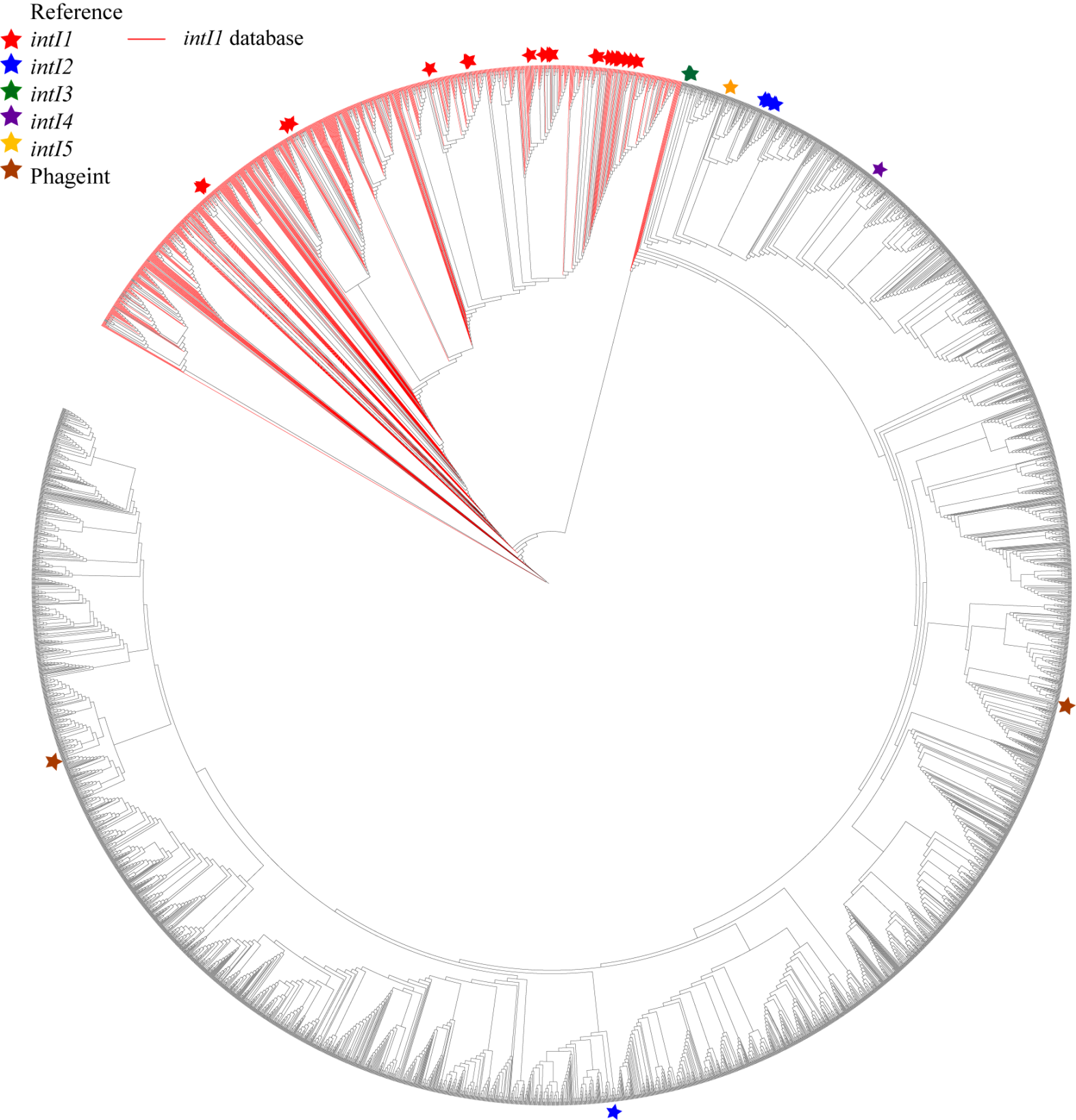


Figure S1. Phylogenetic tree of the integron-integrase database. The clades of integron-integrases were colored by the reference integron-integrases for classification into different classes. The *intI1* sequences (red clade) were extracted to construct the subset database of *intI1* database.


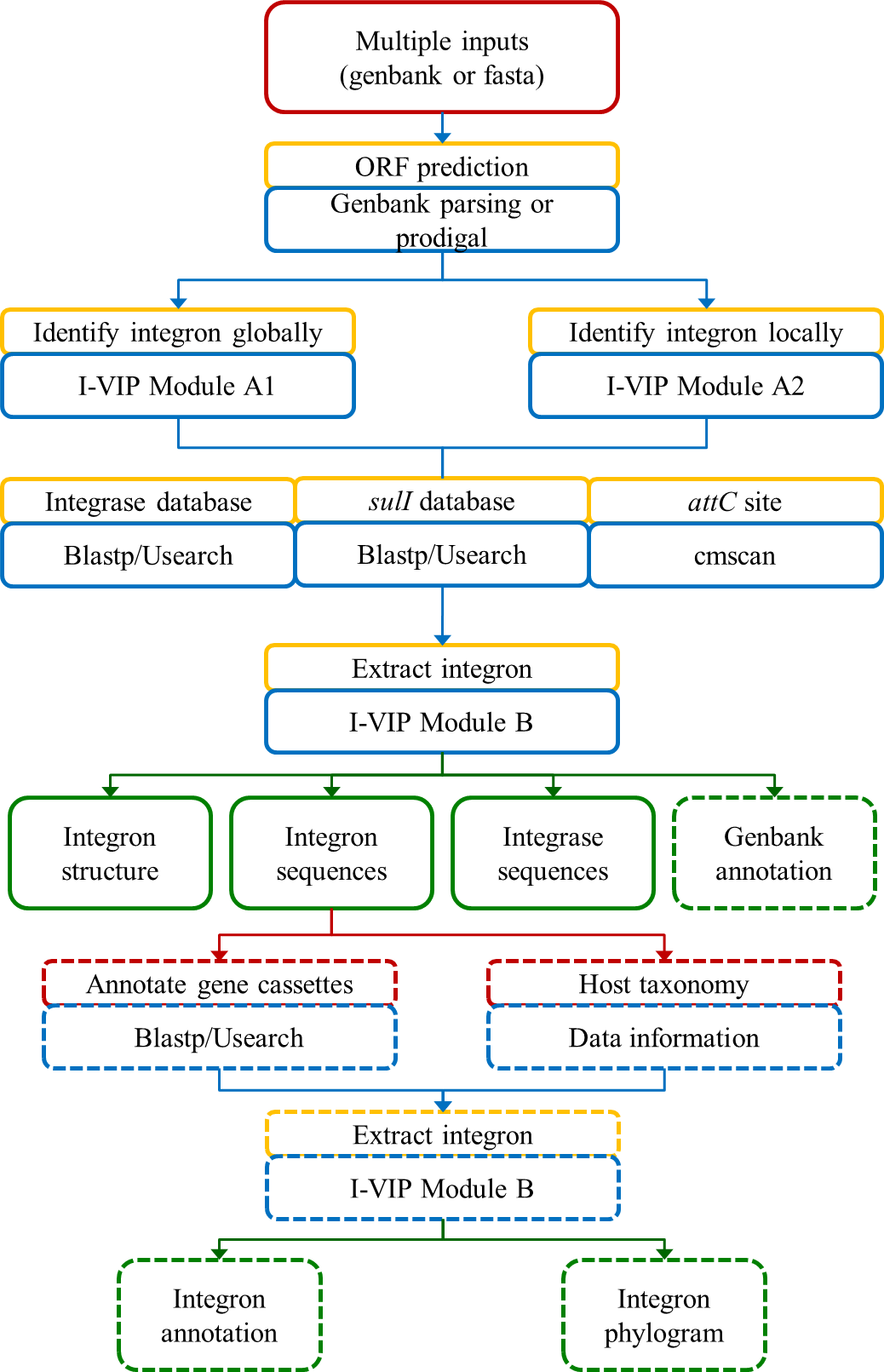


Figure S2. The technical flow of I-VIP pipeline developed in this study.

Figure S3 Structures of integrons of Types A to E.


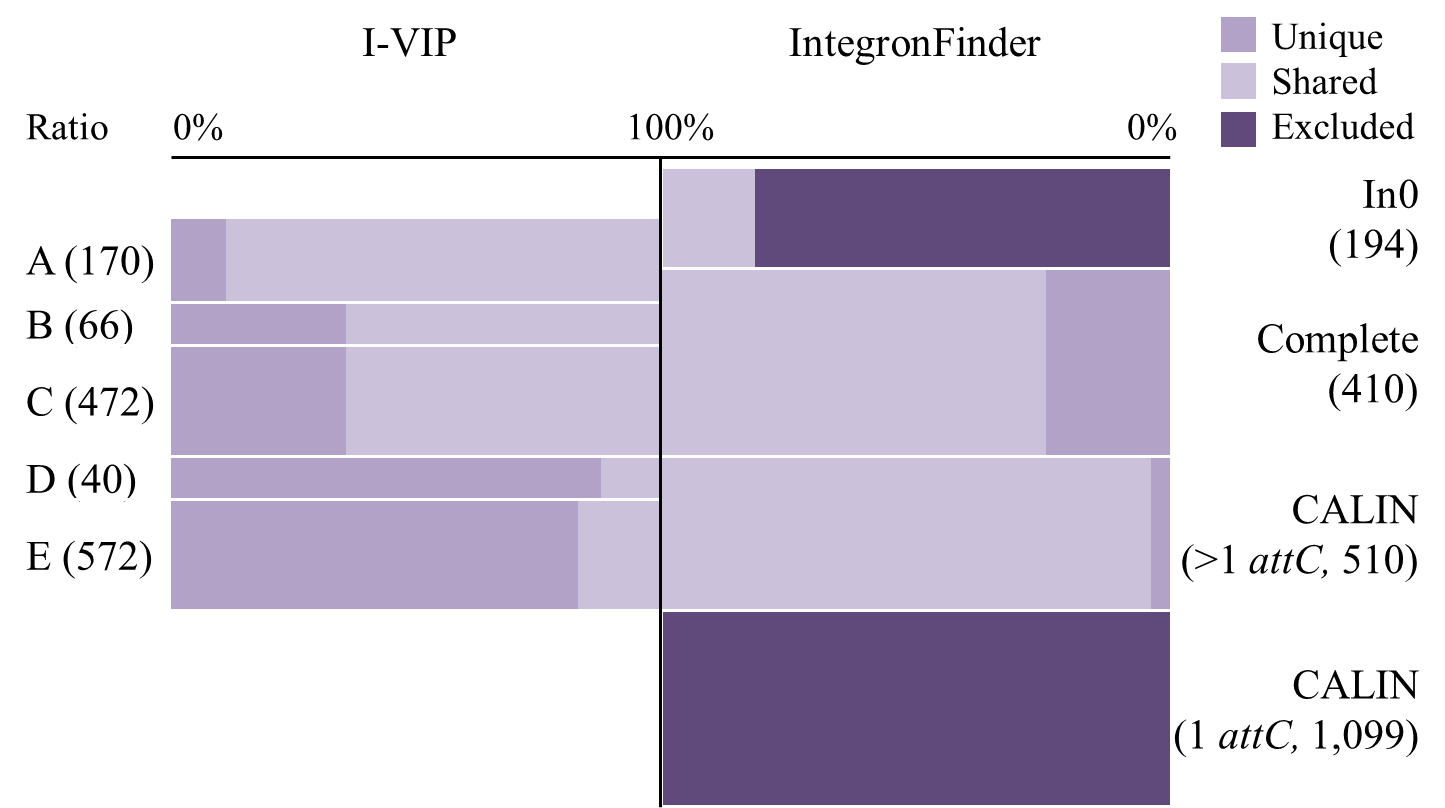


Figure S4. Comparison the integrons identified by I-VIP and IntegronFinder from a set of 5,436 complete genomes. The In0 (single integrases) and CALIN (single *attC* sites) identified by IntegronFinder were not defined as integrons by I-VIP, and were strictly excluded. **attC* sites were missed in these integrons.


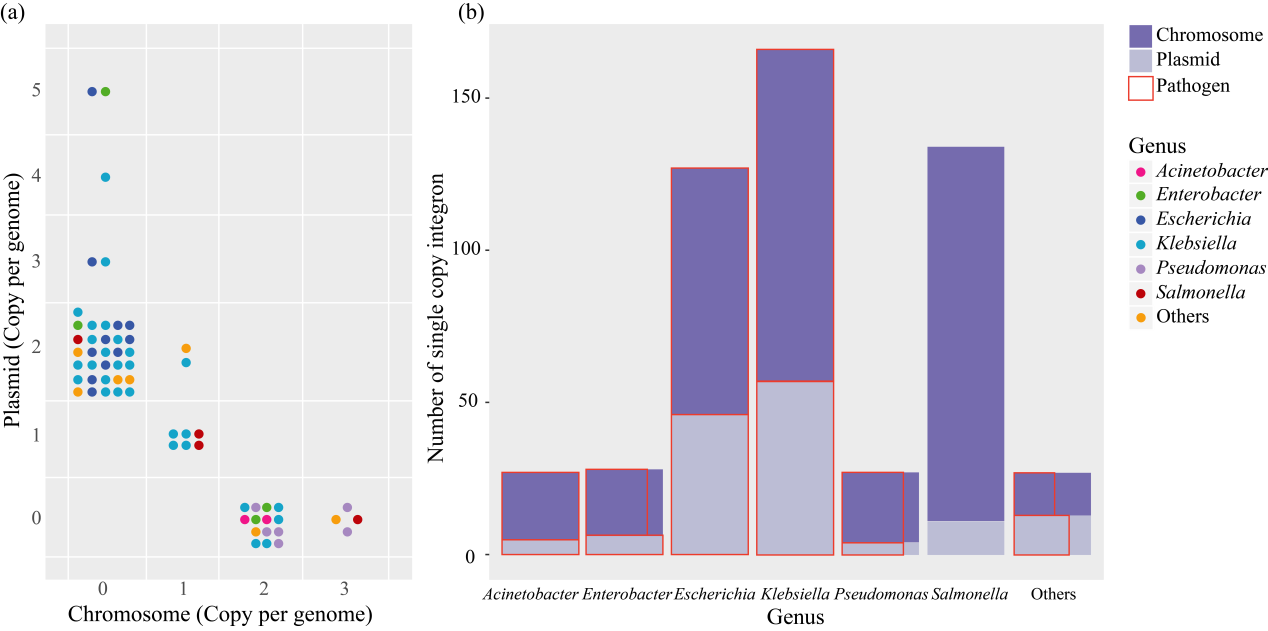


Figure S5. Copy number of integron per genomes. (a) Multiple copies of integrons on the chromosome or plasmid of one genome. (b) Single copy of integrons on the chromosome or plasmid of one genome.

Table S1. Coverage of the *intI1* database constructed in this study and two previous *intI1* databases[1, 13] against all complete class I integron sequences from INTEGRALL database[12].

| Databases (Totally No.) | *intI1* database [13] | intI_Cterm database [1] | *intI1* database in this study | Integron-integrase database in this study |
| --- | --- | --- | --- | --- |
| INTEGRALL database (1,011) | 23.5% (238) | 26.3% (266) | 40.7% (411) | 49.1% (496) |
| INTEGRALL database  with *sulI* (256) | 64.5% (165) | 68.8% (176) | 89.5% (229) | 90.6% (232) |

Table S2. The lists of true positive (TP) and false positive (FP) keywords for the integron-integrase database construction.

| True positive (TP) keyword | False positive (FP) keyword |
| --- | --- |
| *intI1* | integrase catalytic |
| *intI1*delta2 | integrase core |
| delta*intI1* | integrase fusion |
| truncated*intI1* | tnia |
| intsgi | chaperone |
| bla*intI1* | groel |
| truncated*intI1*delta |  |
| *intI1*delta |  |
| *intI1*delta |  |
| int |  |
| int1 |  |
| inti |  |
| *intI1*delta1 |  |
| intidelta1 |  |
| uniprotkb/swiss-prot:p62590 |  |
| integrase |  |
| integrase1 |  |
| integronintegrase |  |
| classiintegronintegrase |  |
| intl1 |  |
| *intI1*integrase |  |
| cog0582integrase |  |
| *intI1* |  |
| phagefamilyintegrase |  |
| DNAintegrase |  |
| integrase |  |
| DNAintegrase |  |
| delta-*intI1* |  |
| truncatedtype1integrase |  |
| integraseclass1 |  |
| classiintegronintegrase |  |
| classisuli-typeintegron |  |
| integrase1 |  |
| integronintegrase |  |
| integronintegrase*intI1* |  |
| *intI1*integrase |  |
| phageintegrase |  |
| phageintegrasefamilyprotein |  |
| phagefamilyintegrase |  |

Table S3. The taxonomic information and accession number of the collection of 73,655 complete and draft bacterial genomes used in this study. The potential pathogenicity was labeled for all bacterial genomes[15].

Table S4. Comparison the integrons identified by I-VIP and IntegronFinder from a set of 5,436 complete genomes. The In0 (single integrases) and CALIN (single *attC* sites) identified by IntegronFinder were not defined as integrons by I-VIP, and were strictly excluded. **attC* sites were missed in these integrons.

| I-VIP | | | | | | IntegronFinder | | | | | |
| --- | --- | --- | --- | --- | --- | --- | --- | --- | --- | --- | --- |
| Integron Type | Total No. | Shared integrons | Integrons missed by IntegronFinder | | | Integron Type | Total No. | Shared integrons | Integrons missed by I-VIP | | |
|  |  |  | Missing integrases | Missing *attC* | Missing integrons |  |  |  | Missing integrases | Missing *attC* | Excluded by I-VIP |
| Type A | 170 | 153 | 17 | 0 | 0 | Complete | 410 | 311 | 99 | 0 | 0 |
| Type B | 66 | 38 | 28 | 0 | 0 |  |  |  |  |  |  |
| Type C | 472 | 154 | 249 | 0 | 69 |  |  |  |  |  |  |
| Excluded by I-VIP | | | | | | In0 | 194 | 34* | 0 | 0 | 160 |
| Type D | 40 | 34 | 0 | 0 | 6 | CALIN | 510 | 500 | 0 | 10 | 0 |
| Type E | 572 | 466 | 0 | 84 | 22 | (>1 *attC*) |  |  |  |  |  |
| Excluded by I-VIP | | | | | | CALIN  (1 *attC*) | 1099 | 0 | 0 | 0 | 1099 |

Table S5. The host information (accession number, microorganism name, assembly level and possible pathogenicity[15]) and integron information (type, gene cassettes structure and annotation, number of ARGs, position on the genomes) were listed for 2,440 integrons identified in WGD and 537 integrons identified in plasmid database.

Table S6. Summary of phylogenetic distribution of integrons (Types A to E) harbored by different taxonomic levels in the collection of 73,655 complete and draft bacterial genomes. Table S7. Summary of phylogenetic distribution of integrons (A to E Types) harbored by different taxonomic levels in the collection of 73,655 complete and draft bacterial genomes. The integron copy number was normalized against the species level, and those pathogenic and non-pathogenic strains within one species were specifically differentiated (in table species). The potential pathogenicity of bacterial species was obtained by merging the pathogenicity of genomes [15]. Phy_sp. The number of species in a phylum. Pre%A, the pathogenic prevalence of host species carrying Type A integrons = No. of pathogenic species carrying Type A integrons / Total No. of species carrying Type A integrons. Pre%Total, the pathogenic prevalence of all species = No. of pathogenic species / Total No. of species.

Table S8. The summary of the occurrences of all the ARG phenotypes in all 2,977 integrons (Types A to E) identified in bacterial whole genome database (WGD) and plasmid database. Abbreviations, Gc, gene cassettes, MLS, macrolide-lincosamide-streptogramin.

| Database | Type | ARG phenotype | | | | | | | | | |  |
| --- | --- | --- | --- | --- | --- | --- | --- | --- | --- | --- | --- | --- |
|  |  | Aminoglycoside | Beta-Lactam | Chloramphenicol | MLS | Quinolone | Rifamycin | Sulfonamide | Tetracycline | Trimethoprim | None-ARG | Gcs No. |
| Chromosomes | A | 3.E-01 | 4.E-02 | 2.E-02 | 1.E-03 | 7.E-04 | 1.E-02 | 0.E+00 | 0.E+00 | 2.E-01 | 5.E-01 | 1517 |
|  | B | 3.E-01 | 6.E-02 | 8.E-02 | 2.E-03 | 0.E+00 | 1.E-02 | 0.E+00 | 0.E+00 | 1.E-01 | 4.E-01 | 886 |
|  | C | 1.E-03 | 8.E-05 | 8.E-05 | 0.E+00 | 0.E+00 | 0.E+00 | 2.E-04 | 0.E+00 | 1.E-03 | 1.E+00 | 12289 |
|  | D | 7.E-02 | 1.E-02 | 2.E-02 | 2.E-03 | 0.E+00 | 2.E-02 | 1.E-01 | 0.E+00 | 1.E-02 | 7.E-01 | 537 |
|  | E | 5.E-03 | 3.E-03 | 9.E-04 | 0.E+00 | 0.E+00 | 2.E-04 | 0.E+00 | 0.E+00 | 5.E-04 | 1.E+00 | 11579 |
| Plasmids | A | 2.E-01 | 4.E-02 | 4.E-02 | 3.E-03 | 4.E-03 | 2.E-02 | 9.E-02 | 0.E+00 | 9.E-02 | 5.E-01 | 3050 |
|  | B | 2.E-01 | 3.E-02 | 5.E-02 | 1.E-03 | 0.E+00 | 2.E-02 | 0.E+00 | 1.E-03 | 5.E-02 | 6.E-01 | 865 |
|  | C | 1.E-01 | 7.E-02 | 0.E+00 | 0.E+00 | 0.E+00 | 0.E+00 | 0.E+00 | 0.E+00 | 2.E-02 | 8.E-01 | 592 |
|  | D | 1.E-01 | 6.E-02 | 7.E-02 | 4.E-03 | 0.E+00 | 5.E-02 | 2.E-01 | 0.E+00 | 4.E-02 | 5.E-01 | 227 |
|  | E | 2.E-01 | 1.E-01 | 8.E-03 | 0.E+00 | 0.E+00 | 3.E-03 | 0.E+00 | 0.E+00 | 8.E-03 | 7.E-01 | 619 |

Table S9. Coverage of the integron and integron-integrase databases by current available PCR-based primers.

| Database (totally No.) | Coverage | Primers |
| --- | --- | --- |
| Integron database (2,153) | 21.9% (471) | *cass1*/*cass2*[16] |
|  | 0.0% (10) | *attC/attC* (MRG284/MRG285)[17] |
|  | 22.5% (485) | 5’-CS/*qacE* (HS458/HS459)[18] |
|  | 23.6% (509) | 5’-CS/3’-CS[19] |
|  | 20.6% (443) | 5’-CS/*sulI*[19] |
|  | 24.1% (518) | hep58/hep59 |
| *intI1* nucleotide database (3,738) | 26.4% (986) | HS463a/HS464[20] |
|  | 31.2% (1,168) | *intA*[16] |
|  | 0.0% (0) | *intB*[16] |
|  | 68.5% (2,562) | *cass1*[16] |

**Supplementary files**

File S1. The integron-integrase database constructed and curated using bioinformatics pipeline and whole genome analysis in this study, covering 3,384 complete and non-redundant integrases.

**References**

1. Cury J, Jové T, Touchon M, Néron B, Rocha EP: **Identification and analysis of integrons and cassette arrays in bacterial genomes.** *Nucleic acids research* 2016, **44:**4539-4550.

2. Yang Y, Jiang X, Chai B, Ma L, Li B, Zhang A, Cole JR, Tiedje JM, Zhang T: **ARGs-OAP: online analysis pipeline for antibiotic resistance genes detection from metagenomic data using an integrated structured ARG-database.** *Bioinformatics* 2016.

3. Joss MJ, Koenig JE, Labbate M, Polz MF, Gillings MR, Stokes HW, Doolittle WF, Boucher Y: **ACID: annotation of cassette and integron data.** *BMC Bioinformatics* 2009, **10:**118.

4. Nawrocki EP, Eddy SR: **Infernal 1.1: 100-fold faster RNA homology searches.** *Bioinformatics* 2013, **29:**2933-2935.

5. Shannon P, Markiel A, Ozier O, Baliga NS, Wang JT, Ramage D, Amin N, Schwikowski B, Ideker T: **Cytoscape: a software environment for integrated models of biomolecular interaction networks.** *Genome research* 2003, **13:**2498-2504.

6. Li L-G, Xia Y, Zhang T: **Co-occurrence of antibiotic and metal resistance genes revealed in complete genome collection.** *The ISME journal* 2017, **11:**651-662.

7. Sayers EW, Barrett T, Benson DA, Bolton E, Bryant SH, Canese K, Chetvernin V, Church DM, DiCuccio M, Federhen S: **Database resources of the national center for biotechnology information.** *Nucleic acids research* 2012, **40:**D13-D25.

8. Camacho C, Coulouris G, Avagyan V, Ma N, Papadopoulos J, Bealer K, Madden TL: **BLAST+: architecture and applications.** *BMC Bioinformatics* 2009, **10:**421.

9. Edgar RC: **Search and clustering orders of magnitude faster than BLAST.** *Bioinformatics* 2010, **26:**2460-2461.

10. Edgar RC: **MUSCLE: multiple sequence alignment with high accuracy and high throughput.** *Nucleic acids research* 2004, **32:**1792-1797.

11. Price MN, Dehal PS, Arkin AP: **FastTree 2–approximately maximum-likelihood trees for large alignments.** *PloS one* 2010, **5:**e9490.

12. Moura A, Soares M, Pereira C, Leitao N, Henriques I, Correia A: **INTEGRALL: a database and search engine for integrons, integrases and gene cassettes.** *Bioinformatics* 2009, **25:**1096-1098.

13. Ma L, Li A-D, Yin X-L, Zhang T: **The Prevalence of Integrons as the Carrier of Antibiotic Resistance Genes in Natural and Man-Made Environments.** *Environmental Science & Technology* 2017, **51:**5721-5728.

14. Huang YT, Yang J-i, Chrobak M, Borneman J: **PRISE2: Software for designing sequence-selective PCR primers and probes.** *BMC bioinformatics* 2014, **15:**317.

15. Woolhouse ME, Gowtage-Sequeria S: **Host range and emerging and reemerging pathogens.** *Emerging infectious diseases* 2005, **11:**1842.

16. Rosser SJ, Young H-K: **Identification and characterization of class 1 integrons in bacteria from an aquatic environment.** *Journal of Antimicrobial Chemotherapy* 1999, **44:**11-18.

17. Gillings MR, Xuejun D, Hardwick SA, Holley MP, Stokes HW: **Gene cassettes encoding resistance to quaternary ammonium compounds: a role in the origin of clinical class 1 integrons?** *ISME J* 2009, **3:**209-215.

18. Holmes AJ, Gillings MR, Nield BS, Mabbutt BC, Nevalainen K, Stokes H: **The gene cassette metagenome is a basic resource for bacterial genome evolution.** *Environmental microbiology* 2003, **5:**383-394.

19. Levesque C, Piche L, Larose C, Roy PH: **PCR mapping of integrons reveals several novel combinations of resistance genes.** *Antimicrobial agents and chemotherapy* 1995, **39:**185-191.

20. Hardwick SA, Stokes HW, Findlay S, Taylor M, Gillings MR: **Quantification of class 1 integron abundance in natural environments using real-time quantitative PCR.** *FEMS Microbiol Lett* 2008, **278:**207-212.
